# Depression, anxiety, and burnout among medical students and residents of a medical school in Nepal: a cross-sectional study

**DOI:** 10.1186/s12888-020-02645-6

**Published:** 2020-06-15

**Authors:** Nishan Babu Pokhrel, Ramesh Khadayat, Pratikchya Tulachan

**Affiliations:** 1 Tribhuvan University Institute of Medicine, Kathmandu, Nepal; 2 Department of Psychiatry and Mental Health, Tribhuvan University Institute of Medicine, Kathmandu, Nepal

**Keywords:** Anxiety, Burnout, Depression, Developing countries, Medical students, Residents, Stressors, South Asia

## Abstract

**Background:**

Medical students and residents were found to have suffered from depression, anxiety, and burnout in various studies. However, these entities have not been adequately explored in the context of Nepal. We proposed to determine the prevalence of depression, anxiety, burnout, their associated factors, and identify their predictors in a sample of medical students and residents in a Nepalese medical school.

**Methods:**

It was a cross-sectional study with 651 medical students and residents chosen at random between December 2018 and February 2019. The validated Nepali version of Hospital Anxiety and Depression Scale, the Copenhagen Burnout Inventory, and Medical Students’ Stressor Questionnaire were used to assess depression, anxiety, burnout, and stressors respectively. We used univariate and multivariable logistic regression analyses to identify the correlation of predictor variables with depression, anxiety, and burnout.

**Results:**

The overall prevalence of burnout (48.8%; 95% CI 44.9–52.7) and anxiety (45.3%; 95% CI 41.4–49.2) was more than that of depression (31%; 95% CI 27.5–34.7). Burnout and depression were more prevalent in residents than in medical students (burnout: 64.5% vs 37.6%, *P*-value < 0.0001; depression: 33.7% vs 29.1%, *P*-value 0.21). Whereas, medical students were found more anxious than residents (46.3% versus 43.96%, *P*-value 0.55). Academic related stressors caused high-grade stress to participants. Multivariable model for depression significantly showed anxiety, personal burnout, and work-related burnout as risk enhancing correlates; satisfaction with academic performance as a protective correlate. Similarly, the multivariate model for anxiety significantly identified female gender, depression, personal burnout, teaching and learning related stressors, and past history of mental illness as risk enhancing correlates; being satisfied with academic performance, getting adequate sleep, and being a second-year resident as protective correlates. The logistic model for burnout significantly showed being a first-year resident, depression, anxiety, and drive and desire related stressors as positive predictors. None of the variables were identified as significant negative predictors of burnout.

**Conclusions:**

A high prevalence of depression, anxiety, and burnout was seen among medical students and residents. Most of them were stressed with academic-related factors. A strong correlation between teaching and learning-related stressors with depression and anxiety may be a call for an efficient and more student-friendly curriculum.

## Background

Medical education is long, and physically and emotionally demanding. Before entering into the medical school, the mental health of medical students is similar to that of the general population [1–3] or even better [4]. Inside the medical school, they are exposed to various academic and psychosocial stressors [5] thought to be typical of the medical school environment. They are exposed to workload [6–8], academic pressure [9], the need to be seen as a competent clinician [10], sleep deprivation [7], peer competition [7], fear of failure in medical school [7], death and suffering of patient [11], student abuse [12], financial problem [7, 8], etc. In addition to these, they also face personal life events, which are beyond the control of medical school authority like illness, marriage, the birth of a child, and death of family members. Due to the aforementioned stressful events, the mental health of medical students declines as they progress in their medical training [2, 6, 13]. The decline starts right from their first year [14]. These stressors result in their poor academic performance, academic dishonesty, cynicism, substance abuse [15], and serious mental illnesses like depression, anxiety, and burnout*.* The poor health-related quality of life among medical students is contributed mainly by the mental component [16].

The data regarding the status of mental health among Nepalese medical students and residents are sparse, as enough studies have not been conducted here. A few studies on the prevalence of anxiety and depressive disorders among medical students have been done in Nepal which revealed a high prevalence of anxiety and depressive disorders [17–20]. In developing nations like Nepal, medicine is not only looked upon as a career, but also as an opportunity for social advancement. This may result in increased pressure to students as compared to their counterparts in developed nations [21].

Burnout is defined as a psychological syndrome of emotional exhaustion, depersonalization, and reduced personal accomplishment induced by repeated exposure to workplace stressors [22]. Emotional exhaustion is the feeling of extreme fatigue with draining of emotional resources due to repeated exposure to workplace stressors. Depersonalization is the distant attitude towards the job in which the person attempts to keep a distance between oneself and the clients. Reduced personal efficacy implies a feeling of being ineffective in the work accompanied by the feeling of low self-esteem [23]. The components of burnout can also be grouped as personal burnout, work-related burnout, and client-related burnout [24]. Personal burnout [24] refers to the degree of physical and psychological fatigue experienced by the person regardless of his/her work. Work-related burnout [24] refers to the degree of physical and psychological fatigue perceived by the person as related to his/her work. Client-related burnout [24] refers to the fatigue-related to interaction with clients.

Burnout in healthcare workers is a universal concern [25]. Different studies across the world have shown a high prevalence of burnout among medical students [21, 26, 27] and residents [28]. Burnout is found to be prevalent among medical students even prior to the initiation of the clinical years of medical training [29, 30]. It then progressively develops over the course of medical training [31]. A recent meta-analysis has found a high prevalence of burnout in medical and surgical residents [32]. Various factors predict the likelihood of burnout in physicians which are job dissatisfaction [33], huge work intensity, and lack of time off [33]. Physician burnout is linked with an increase in medical errors [34] and reduced quality of patient care [35]. Burnout also increases the risk of suicidal ideation [27, 36]. Burnout is significantly associated with state-anxiety [37] and depression [38, 39]. Neglected burnout also results in other serious consequences such as substance abuse, depression, insomnia, and impaired interpersonal and marital relationship [40].

About a third of medical students in the world suffer from depression [41] which is much higher than the general population (about 3.9–6.6%) [42, 43] and non-medical students (19% in men, 22% in women) [44]. A higher level of depression was reported in first-year residents as well [45]. Depression is found to be significantly associated with chronic sleep deprivation [45], stressful personal life events, and burnout [46]. Burnout and depressive symptoms significantly predicted serious thoughts of dropping out of the medical school [47]. Interestingly, burnout has been described as a form of depression [48]. Burnout and depression have their etiological association with repeated and unresolvable stress. Unresolvable stress has been described as causing depression as well [49].

The prevalence of depression in Nepalese medical students was found to be around 30% and was consistent across all four studies [17–20]. The prevalence of anxiety was found to be around 5.8% [18]. Adhikari et al. [18] attempted to widen the focus into the study of depression, anxiety, suicidal ideation, marijuana use, smoking, and eating disorders while other studies were focused on depression [17, 19, 20], suicidal ideation [50], anxiety disorders [19], and stressors [5]. None of them included residents in their study and none studied the prevalence of burnout either.

Hence, we planned this study to determine the prevalence of depression, anxiety, burnout and their associated factors, and identify their predictors among medical students and residents of a Nepalese medical school.

## Methods

### Study design, setting, and participants

We carried out a descriptive cross-sectional study between December 2018 and February 2019 in Maharajgunj Medical Campus (MMC) located in Kathmandu, Nepal. All the medical students and residents who had spent at least a year in the medical school were considered eligible for the study. Students who were not available during the data collection period were excluded from the study.

### Sample size and sample procedure

Simple random sampling based on the lottery method was carried out to select the participants. The sample size was calculated using the formula:

$$ n=\frac{Z^2p\left(1-p\right)}{E^2}, $$

where, *n* = minimum sample size when population is large, *Z* = confidence level at 95% (standard value of 1.96), *p* = prevalence (taken 0.30 based on 29.9% prevalence of depression in a similar Nepalese study [19]), *E* = allowable error (5% of *p*). For finite population: $n\ \mathrm{finite}=\frac{n}{1+\frac{n}{N}}$,

where *n* = calculated initial sample size using the previous formula, which was 3585 and *N* = finite population number (the total number of medical students and residents of our medical school = 732).

$$ n\ \mathrm{finite}=\frac{3585}{1+\frac{3585}{732}} $$, *n*_finite = 608._

After considering the proportion (*q)* that was expected to refuse to participate or provide inadequate information, the final number of samples to be recruited was

$$ {n}^{\ast }=\frac{n}{1-q}, $$

Where *q* is the proportion of attrition and was taken as 10%. After adjusting for the number of non-responses, the final number of samples was 676.

### Outcome variables

Depression, anxiety, and burnout were classified as the outcome variables. Depression and anxiety were assessed by using the validated Nepali version of Hospital Anxiety and Depression Scale (HADS) [51] which consists of two subscales: namely an anxiety scale (HADS-A) and a depression scale (HADS-D) each with seven items. As an example, the characteristic items are: *“I feel tensed or ‘wound up’”, “I can laugh and see the funny side of things”, “I have lost interest in my appearance”.* All 14 items were rated along a four-point Likert scale according to responses of frequency from zero to three. By adding these up, a sum value for the two scales was obtained. Values from 0 to 7 were considered as normal, 8 to 10 as borderline, and between 11 to 21 as suspicious [52]. In our study, we classified both the borderline and the suspicious groups as having depression. In the same way, we classified the participants as having anxiety and not having anxiety. The internal consistency of the Nepali version of HADS was satisfactory (HADS-A α = 0.76 and HADS-D α = 0.68) [51]. In the current study, the Cronbach’s alpha reliability coefficient was good (HADS-A α = 0.74 and HADS-D α = 0.73).

Burnout was assessed by using Copenhagen Burnout Inventory (CBI) [24] consisting of three sub-scales measuring specifically personal burnout (6 items), work-related burnout (7 items), and client-related burnout (6 items). We changed the term ‘client’ into ‘patient’; ‘work’ into ‘work/study’, and accordingly replaced them in the questionnaire. So, ‘client-related’ burnout became ‘patient-related’ burnout, and ‘work-related’ burnout became ‘work/study-related’ burnout. Twelve items were rated along a five-point Likert scale according to responses of frequency from ‘100 (always)’ to ‘0 (never/almost never)’. The remaining seven items, however, rate the response according to an intensity which ranges from ‘to a very low degree’ to ‘to a very high degree’ [24]. But, an item in the work-related burnout subscale required inverse scoring and the item was: *“do you have enough energy for family and friends during leisure time?”* Typically, items in the scale were: “*how often do you feel worn out?”, “do you feel burnt out because of your work?”, “do you find it hard to work with patients?”.* The level of burnout was classified according to the scores obtained. A score of zero to 50 implies “no/low”, 50 to 74 implies “moderate’, 75 to 99 implies ‘high’, and a score of 100 implies ‘severe’ burnout. All the items had high internal consistency, and were straightforward and related to the relevant subscale. In our study, the Cronbach’s alpha reliability coefficients of the three CBI subscales were high (personal burnout α = 0.79; work-related burnout α = 0.87; and patient-related burnout = 0.85). Burnout was defined if any one of the personal, work-related or patient-related burnout was present in a student.

### Independent variables

#### Socio-demographic factors

Socio-demographic factors included age, gender, religion, nationality, socioeconomic class, current residence, and the year of training in medical school. The variable ‘age’ was grouped into four age-groups starting from 18 years with increments of 5 years (group 1 = 18–24 years, group 2 = 25–29 years, group 3 = 30–34 years, group 4 = 35–39 years). The socioeconomic class was measured using the modified Kuppuswamy’s Socioeconomic Status scale in the context of Nepal [53]. The scale consisted of three criteria namely educational, occupational, and economic (monthly family income) based on which a score was given. According to the sum of these three scores, the socioeconomic class was determined according to the classification (26–29 = Upper, 16–25 = Upper middle, 11–15 = Lower middle, 5–10 = Upper Lower, < 5 = Lower).

#### Behavioral and clinical factors

Behavioral factors included relationship status, substance use, involvement in extracurricular activities, and sleep hours. Substance use by a person was defined when he/she uses either alcohol, cigarette, or marijuana. The variable ‘sleep hours’ was divided into two groups: adequate sleep and inadequate sleep by using a cut-off of 7 h [54]. Clinical factors included stressors, satisfaction with career choice, satisfaction with academic performance, previous history of mental health problems, family history of mental health problems, and current treatment regarding mental health issues.

The stressors were identified by using the Medical Students’ Stressor Questionnaire-20 (MSSQ-20) [55]. MSSQ-20 consisted of 20 items representing the six stressor domains which were: academic related stressors (ARS), intrapersonal and interpersonal related stressors (IRS), teaching and learning-related stressors (TLRS), social related stressors (SRS), drive and desire related stressors (DRS), and group activities related stressors (GARS). All 20 items were rated along a five-point Likert scale according to the intensity from ‘zero (causing no stress at all)’ to ‘four (causing severe stress)’. The mean score of each of the domain was calculated, and thus the severity of the stress caused by that domain was assessed according to the classification (0-1= Mild, 1.01-2= Moderate, 2.01-3= High and 3.01-4= Severe). High-grade score in a particular stressor group indicated that it caused a lot of stress, disturbed emotions, and mildly compromised daily activities. It was a valid and reliable instrument with high internal consistency as shown by Cronbach’s alpha coefficient value of 0.95. In the current study, the reliability coefficient was high (α= 0.91). The Cronbach’s alpha for each stressor domain was also high (ARS α= 0.87, IRS α= 0.89, TLRS α= 0.77, SRS α= 0.74, DRS α= 0.70, GARS α= 0.76). Each of the stressors ARS, IRS, TLRS, SRS, DRS, and GARS was grouped into two groups: ‘absent’ and ‘present’. The ‘absent’ group included those participants who felt only the mild form of those stressors while the ‘present’ group included all other participants feeling the moderate, severe, and high degree of those stressors.

### Methods of data collection

For the purpose of data collection, the aim of the study was briefly described, and doubts of the participants regarding the study were cleared by the investigators. Participants were requested to choose the item in the questionnaire that was closest to what they have been feeling in the past week, to minimize recall bias. Following this, the questionnaire form was distributed to them. Questionnaire form contained questions regarding socio-demographic, behavioral, and clinical characteristics of the participants along with the scales for measuring depression, anxiety, burnout, and stressors in the participants. We used reliable and validated instruments to minimize information bias. Our survey questionnaire is provided as an Additional file 1.

### Statistical analysis

R software (version 3.5.3) [56] and various R packages were used for statistical analyses. ‘G-models’ package [57] was used to construct a cross-table, ‘caret’ [58] and ‘caTools’ package [59] for multivariable logistic regression analyses, ‘rcompanion’ [60] for calculating Cox and Snell, and Nagelkerke pseudo R squared, ‘lmtest’ [61] for likelihood ratio test, ‘ROCR’ [62] and ‘Metrics’ [63] for calculating AUC and plotting ROC curve, ‘ResourceSelection’ [64] for Hosmer Lemeshow test, ‘survey’ [65] for Wald test, ‘corrplot’ [66] for plotting the contingency table, and ‘ggplot’ [67] for making bar plots.

A total number of 43 variables (numerical-34, categorical-9) from 19 observations were missed. The missing data in a numerical variable were replaced by averages and that of a categorical variable by modes. Descriptive statistics were used for sociodemographic, behavioral, and clinical variables. We used an alpha level of 0.05 as a cut-off point for statistical significance. Univariate analysis was used to find the association of depression, anxiety, and burnout with independent variables. The multivariable logistic regression analysis was used to determine the predictors of depression, anxiety, and burnout. We tested for multicollinearity by calculating variance inflation factor (VIF) score for each variable in the predictor models using the “vif” function of “car” package [68]. We set the cut-off VIF score of 10 and found two variables (“age” and “year”) having higher VIF scores. Thus, we removed a variable (“age”) and rechecked for the collinearity among the remaining variables. As we found no correlation among the remaining variables, we reconstructed the logistic models using the variables except “age”. The stepwise logistic regression using backward elimination method was performed using R software. The scripts used while preparing these models in R software will be available from the author upon reasonable request.

The model with the lowest AIC (Akaike Information Criterion) value was selected as the best-fitted model. Likelihood ratio test and Hosmer-Lemeshow tests were done to test for the goodness of fit of the model. The Cox and Snell, and Nagelkerke pseudo R squared represented the proportion of variance in the outcome variable that was explained by the variables in the model. Tests of individual predictors were done to assess the relative importance of those variables in the model, like Variable Importance using ‘caret’ package [58] and Wald test using ‘survey’ package [65]. Validation of predicted values was done by constructing ROC curve using ‘ROCR’ [62] and ‘Metrics’ [63] packages.

### Ethical considerations

Ethical clearance was obtained from the Institutional Review Committee of the Institute of Medicine (Reference number- 305/075/076). A cover letter consisting of informed consent was attached with each questionnaire, which included a description of the study and participants’ rights to decline altogether or to leave the questions answered. The consent was implied through the completion of the questionnaire. The name, address, or signature of the participant were not included in the questionnaire to keep the identity of the participant anonymous. Participants did not receive any incentives or financial compensation for participating in the study.

## Results

### Participants

The total number of medical students and residents studying in the Maharajgunj Medical Campus was 732. The calculated sample size required for the study with an allowable error of 5% of *p* was 676. A total of 651 students returned the questionnaire form with a non-response rate of 3.7%. Among them, 495 (76%) were males and 156 (24%) were females. The third-year medical students had the highest participation (71 out of 77, 93.4%) whereas, the interns had the lowest (47 out of 60, 78.3%). Interns were those medical students who passed their final year and were doing an internship. Similarly, first-year residents had greater participants, 97 (14.9%) compared to second and third-year residents.

### Characteristics of study participants

The socio-demographic profile of the participants is presented in Table 1. The mean age was 25 years. Most of the participants belonged to the age category 18–24 years (314, 48.2%), whereas only 4 (0.6%) participants were over 35 years of age. Most of the participants were Hindu (611, 93.9%). The number of Nepalese participants was 566 (86.9%),; remaining others were Indian, Sri Lankan, and Maldivian. Majority of the participants were single (392, 60.3%) and only 135 (20.8%) were married. Most of the participants belonged to upper-middle socioeconomic class (302, 46.5%), followed by upper (215, 33.1%), lower-middle (121, 18.6%), and upper-lower (11, 1.7%) socio-economic classes. Only a single participant was residing in the hospital quarter, while a majority (240, 36.9%) were living in rented rooms.

**Table 1:** Socio-demographic profile of the participants, *N* = 651

Socio-demographic features	***n,*** (%)
Male	495 (76)
Mean age (*SD*)	25 (4)
Medical students	378 (58.1)
First-year	66 (10.1)
Second-year	68 (10.4)
Third-year	71 (10.9)
Fourth-year	63 (9.7)
Fifth-year	63 (9.7)
Interns	47 (7.2)
Clinical medical students	244 (37.63)
Non-clinical medical students	134 (20.58)
Residents	273 (41.9)
First-year	97 (14.9)
Second-year	93 (14.3)
Third-year	83 (12.7)
Clinical residents	245 (37.63)
Non-clinical residents	28 (4.3)
18–24 years	314 (48.2)
25–29 years	211 (32.4)
30–34 years	122 (18.7)
35–39 years	4 (0.6)
Religion	
Hindu	611 (93.9)
Buddhist	7 (1.1)
Muslim	28 (4.3)
Christian	2 (0.3)
Sikh	2 (0.3)
Yumanism	1 (0.2)
Nationality	
Nepalese	566 (86.9)
Indian	67 (10.3)
Sri Lankan	6 (0.9)
Maldivian	12 (0.18)
Relationship status	
Married	135 (20.8)
Non-married partner	122 (18.8)
Single	392 (60.3)
Divorced	1 (0.2)
Socio-economic status	
Upper	215 (33.1)
Upper-middle	302 (46.5)
Lower-middle	121 (18.6)
Upper-lower	11 (1.7)
Lower	0 (0)
Current residence	
Home	224 (34.4)
Rented room	240 (36.9)
Hostel	185 (28.4)
Relative	1 (0.2)
Quarter	1 (0.2)

Table 2 summarizes the behavioral and clinical characteristics of the participants. The number of participants having the thoughts of suicide and thoughts of dropping out of medical school were 41 (6.3%) and 79 (12.1%) respectively. Similarly, 35 (5.4%) participants had a previous history of mental disorders, 5 (0.8%) were currently receiving treatment for mental illnesses, and 55 (8.4%) participants had a family history of mental problems. Participants usually slept for an average of 7 h. The number of participants who were not satisfied with their career choice was 145 (22.31%). A large number of participants, 307 (47.23%) were not satisfied with their academic performance. Only 221 (34%) of participants were involved in extracurricular activities. The number of participants who drank alcohol, smoked cigarettes, or smoked marijuana was 393 (60.4%), 241 (37.1%), and 100 (15.4%) respectively. Substance use was found in 398 (61.1%) of participants.

**Table 2:** Clinical and behavioral characteristics of the participants

Features	***n*** (%, 95 CI) ^a^
Suicidal thoughts	41 (6.3, 4.6–8.4)
Thoughts of dropping out	79 (12.1, 9.7–14.9)
Previous history of mental disorders	35 (5.4, 3.8–7.4)
Currently receiving treatment for mental health disorders	5 (0.8, 0.2–1.8)
Family history of mental health problems	55 (8.4, 6.4–10.9)
Sleep hours, mean (*SD*)	7 (1)
Adequate, *n* (%)	441 (67.7, 64–71.3)
Inadequate	210 (32.3, 28.7–36)
Not satisfied with career choice	145 (22.31, 19.1–25.7)
Not satisfied with academic performance	307 (47.23, 43.2–51.1)
Involvement in extracurricular activities	221 (34, 30.3–37.7)
Alcohol	393 (60.4, 56.5–64.1)
Cigarette	241 (37.1, 33.3–40.9)
Marijuana	100 (15.4, 12.7–18.4)
Substance use	398 (61.14, 57.3–64.9)
Depression	202 (31, 27.5–34.7)
Anxiety	295 (45.3, 41.4–49.2)
Burnout	318 (48.8, 44.9–52.7)
Personal burnout	266 (40.8, 37.1–44.7)
Work-related burnout	210 (32.3, 28.7–36)
Patient-related burnout	105 (16.1, 13.4–19.2)

*Note.*^a^ 95% CI, 95% Confidence Interval

**Fig. 1:**
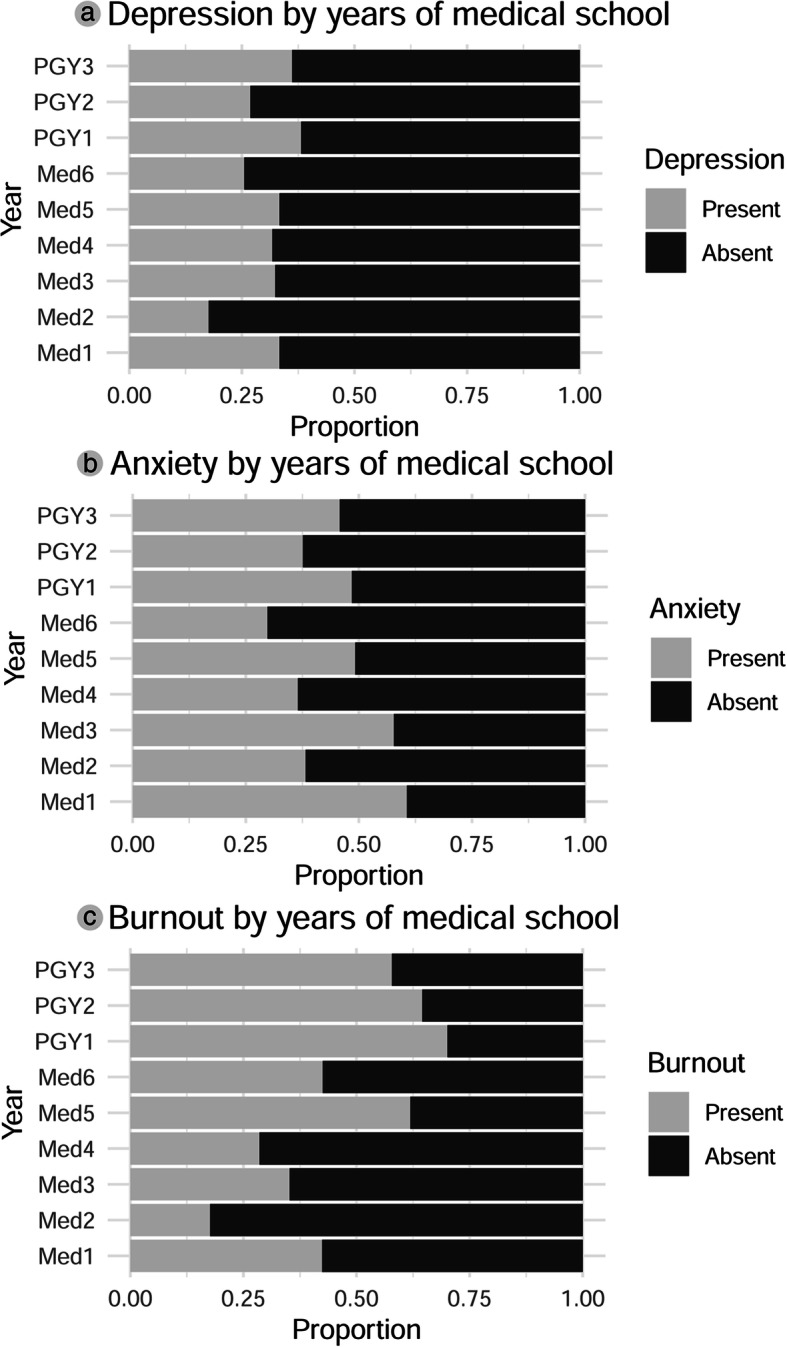
Depression (**a**), anxiety (**b**) and burnout (**c**) according to the years of training of participants. (Med1-Med5: First-year to final year medical students, Med6: Interns, PGY1–3: First year to third-year residents)

Depressive features were seen in 202 (31%) participants, and the features of anxiety were found in 295 (45.3%) participants. A huge number (318, 48.8%) of participants suffered at least one of personal, work-related, or patient-related burnout with greater proportion, 266 (40.8%) of them suffering from personal burnout. Mean scores on the HADS-A, HADS-D, CBI-Personal, CBI-Work/study, and CBI-Patient subscales were 7.39 (standard error of the mean *SE* = 0.13), 6.08 (*SE* = 0.14), 47.93 (*SE* = 0.66), 42.61 (*SE* = 0.72), and 35.02 (*SE* = 0.75) respectively. Figure 1 compares the prevalence of depression, anxiety, and burnout by years of training of participants.

Among the six dimensions of stressors mentioned in Table 3, the ARS group of stressors caused high stress to participants (mean score-2.05) compared to other stressors. Almost all other stressor groups predominantly caused a moderate level of stress to most of the participants. The association of an individual group of stressors with outcome variables (Table 4) showed that depression, anxiety, and burnout were significantly less in the absence of these stressors.

**Table 3:** Domain-wise distribution of stress among the study population

Domains of stressors	Mild, ***n*** (%)	Moderate, ***n*** (%)	High, ***n*** (%)	Severe, ***n*** (%)	Mean score	Grade
ARS	93 (14.3)	264 (40.6)	213 (32.7)	81 (12.4)	2.05	High
IRS	231 (35.5)	236 (36.3)	141 (21.7)	43 (6.6)	1.58	Moderate
TLRS	261 (40.1)	280 (43)	83 (12.8)	27 (4.1)	1.47	Moderate
SRS	262 (40.2)	275 (42.2)	97 (14.9)	17 (2.6)	1.45	Moderate
DRS	367 (56.4)	201 (30.9)	62 (9.5)	21 (3.2)	1.2	Moderate
GARS	225 (34.6)	263 (40.4)	133 (20.4)	30 (4.6)	1.45	Moderate

*Note. ARS* Academic Related Stressor, *IRS* Interpersonal and intrapersonal Related stressor, *TLRS* Teaching and Learning Related Stressor, *SRS* Social Related Stressor, *DRS* Drive and Desire Related Stressor, *GARS* Group Activities Related Stressor

**Table 4:** Association of stressors with depression, anxiety, and burnout

Stressor groups	Depression, χ^**2**^, ***P-***value ***n*** (%)	Anxiety, χ^**2**^, ***P-***value ***n*** (%)	Burnout, χ^**2**^, ***P-***value ***n*** (%)
ARS	9.69, 0.002	13.19, *P* < 0.001	15.25, *P* < 0.0001
Present (*n =* 558)	186 (33.3)	269 (48.2)	290 (52)
Absent (*n =* 93)	16 (17.2)	26 (28)	28 (30.1)
IRS	8.72, 0.003	15.18, *P* < 0.0001	15.26, *P* < 0.0001
Present (*n =* 420)	147 (35)	214 (51)	229 (54.5)
Absent (*n =* 231)	55 (23.8)	81 (35.1)	89 (38.5)
TLRS	26.87, *P <* 0.0001	32.11, *P* < 0.0001	55.32, *P* < 0.0001
Present (*n =* 390)	151 (38.7)	212 (54.4)	237 (60.8)
Absent (*n =* 261)	51 (19.5)	83 (31.8)	81 (31)
SRS	7.93, 0.005	9.04, 0.003	8.27, 0.004
Present (*n =* 389)	137 (35.2)	195 (50.1)	208 (53.5)
Absent (*n =* 262)	65 (24.8)	100 (38.2)	110 (42)
DRS	19.54, *P* < 0.0001	7.55, 0.006	63.17, *P* < 0.0001
Present (*n =* 284)	114 (40.1)	146 (51.4)	189 (66.5)
Absent (*n =* 367)	88 (24)	149 (40.6)	129 (35.1)
GARS	32.12, *P* < 0.0001	29.78, *P* < 0.0001	37.03, *P* < 0.0001
Present (*n* = 426)	164 (38.5)	226 (53.1)	245 (57.5)
Absent (*n =* 225)	38 (16.9)	69 (30.7)	73 (32.4)

*Note. ARS* Academic Related Stressor, *IRS* Interpersonal and intrapersonal Related stressor, *TLRS* Teaching and Learning Related Stressor, *SRS* Social Related Stressor, *DRS* Drive and Desire Related Stressor; *GARS* Group Activities Related Stressor

all *P*-values were obtained by using the Chi-squared test.

### Independent correlates of depression-related symptoms

Using univariate analysis, depression was found to be significantly correlated with many variables (Table 5). Further, we used logistic regression to assess the impact of available variables on the likelihood that the study participant would suffer from depressive symptoms. The final model (Table 6) was statistically significant (*P* <  0.001), indicating that the model was able to distinguish whether the study participant was suffering from depression or not. The final model explained between 18.4% (Cox and Snell R squared) and 25.5% (Nagelkerke R squared) of the variance in depression and correctly classified 84% of the cases. The logistic regression model shows that depression was significantly predicted by the presence of anxiety (*OR* 4.13; 95% CI 2.64–6.57), personal burnout (*OR* 1.71; 95% CI 1.03–2.83), and work-related burnout (*OR* 1.78; 95% CI 1.05–3.01). A student who was satisfied with his/her academic performance (*OR* 0.51; 95% CI 0.33–0.80) was less likely to get depressed.

**Table 5:** Univariate analysis for depression, anxiety, and burnout, and selected demographic variables

Participant’s characteristics	Depression χ^**2**^, ***P-***value ***n*** (%)	Anxiety χ^**2**^, ***P-***value ***n*** (%)	Burnout χ^**2**^, ***P-***value ***n*** (%)
Gender	7.28, 0.007	16.93, *P* < 0.0001	3.93, 0.05
Male (*n* = 495)	140 (28.3)	202 (40.8)	231 (46.7)
Female (*n* = 156)	62 (39.7)	93 (59.6)	87 (55.8)
Trainee type	1.57, 0.21	0.35, 0.55	45.92, *P* < 0.0001
Medical Students (*n* = 378)	110 (29.1)	175 (46.3)	142 (37.6)
Residents (*n* = 273)	92 (33.7)	120 (44)	176 (64.5)
Years in training	10.81, 0.21	21.57, 0.006	77.63, *P* < 0.0001
Medical student			
First-year (*n* = 66)	22 (33.3)	40 (60.6)	28 (42.4)
Second-year (*n* = 68)	12 (17.7)	26 (38.2)	12 (17.6)
Third-year (*n* = 71)	23 (32.4)	41 (57.8)	25 (35.2)
Fourth-year (*n* = 63)	20 (31.8)	23 (36.5)	18 (28.6)
Fifth-year (*n* = 63)	21 (33.3)	31 (49.2)	39 (61.9)
Interns (*n* = 47)	12 (25.5)	14 (29.8)	20 (42.6)
Residents			
First-year (*n* = 97)	37 (38.1)	47 (48.5)	68 (70.1)
Second-year (*n* = 93)	25 (26.9)	35 (37.6)	60 (64.5)
Third-year (*n* = 83)	30 (36.1)	38 (45.8)	48 (57.8)
Clinical or non-clinical med student or resident	6.57, 0.09	1.10, 0.78	58.78, *P* < 0.0001
Clinical med std (*n* = 244)	76 (31.2)	109 (44.7)	102 (41.8)
Non-clinical med std (*n* = 134)	34 (25.4)	66 (49.3)	40 (29.9)
Clinical res (*n* = 245)	87 (35.5)	108 (44.1)	165 (67.3)
Non-clinical res (*n* = 28)	5 (17.9)	12 (42.9)	11 (39.3)
Age category	0.13 ^a^	0.75 ^a^	*P* < 0.0001 ^a^
18–24 years (*n* = 314)	88 (28)	148 (47.1)	109 (34.7)
25–29 years (*n* = 211)	65 (30.8)	92 (43.6)	130 (61.6)
30–34 years (*n* = 122)	48 (39.3)	54 (44.3)	76 (62.3)
35–39 years (*n* = 4)	1 (25)	1 (25)	3 (75)
Satisfied with academic performance	43.2, *P* < 0.0001	50.1, *P* < 0.0001	12, *P* < 0.001
Satisfied (*n* = 344)	68 (19.8)	111 (32.3)	146 (42.4)
Dissatisfied (*n =* 307*)*	134 (43.6)	184 (59.9)	172 (56)
Satisfied with career choice	28, *P* < 0.0001	22.9, *P* < 0.0001	28.2, *P* < 0.0001
Satisfied (*n* = 506)	131 (25.9)	204 (40.3)	219 (43.3)
Dissatisfied (*n =* 145*)*	71 (49)	91 (62.8)	99 (68.3)
Extracurricular involvement	*P* < 0.001^a^	0.12^a^	36.9, *P* < 0.0001
Always (*n* = 56)	16 (28.6)	31 (55.4)	15 (26.8)
Often (*n* = 166)	36 (21.7)	65 (39.2)	70 (42.2)
Sometimes (*n* = 240)	70 (29.2)	112 (46.7)	111 (46.3)
Rarely (*n* = 177)	75 (42.4)	79 (44.6)	111 (62.7)
Never (*n =* 12)	5 (41.7)	8 (66.7)	11 (91.7)
Adequacy of sleep	2.9, 0.09	1.7, 0.19	22.7, *P* < 0.0001
Adequate (*n* = 441)	127 (28.8)	192 (43.5)	187 (42.4)
Inadequate (*n* = 210)	75 (35.7)	103 (49)	131 (62.4)
Substance use	0.37, 0.54	1.41, 0.23	6.29, 0.01
Using (*n* = 398)	127 (31.9)	173 (43.5)	210 (52.8)
Not using (*n* = 253)	75 (29.6)	122 (48.2)	108 (42.7)

*Note. med std* medical student, *res* resident; ^a^: *P*-values calculated from Fisher’s exact test; all other *P*-values were obtained by using the Chi-squared test.

**Table 6:** Final model of a multivariable logistic regression analysis predicting depression among medical students and residents

Predictor	β	***SE***	***P***	***OR*** (95% CI)
Intercept	−1.690	0.240	< 0.001	0.18 (0.114–0.291)
Anxiety	–	–	–	–
Absent ^a^	–	–	–	–
Present	1.412	0.232	< 0.001	4.129 (2.639–6.574)
Personal burnout	–	–	–	–
Absent ^a^	–	–	–	–
Present	0.534	0.258	0.04	1.706 (1.026–2.831)
Work-related burnout	–	–	–	–
Absent ^a^	–	–	–	–
Present	0.576	0.267	0.03	1.780 (1.054–3.009)
Satisfied with academic performance	–	–	–	–
Dissatisfied ^a^	–	–	–	–
Satisfied	−0.666	0.224	0.003	0.514 (0.330–0.796)
Test	χ^2^	*df*	*P*	
Hosmer and Lemeshow test	4.09	8	0.85	
Likelihood ratio test	4	97.68	*<* 0.001	

*Note.*^a^referent for dummy coding. The coefficient (β) in the model indicates the direction (positive or negative) and the significance of the relationship to the outcome variable (depression). Null deviance: 612.43 on 479 degrees of freedom (*df*), Residual deviance: 514.75 on 475 *df*, AIC (Akaike Information Criterion): 524.75, AUC (Area Under Receiver Operating Characteristic Curve): 0.71

### Independent correlates of anxiety-related symptoms

Many variables which were associated with anxiety-related symptoms were shown by the univariate analysis (Table 5). Multivariable logistic regression analysis showed those variables which correlated independently and strongly with anxiety-related symptoms. The final model (Table 7) was statistically significant (*P* = 0.049), indicating that the model was able to distinguish whether the study participant was suffering from anxiety or not. The final model explained between 23.4% (Cox and Snell R squared) and 31.3% (Nagelkerke R squared) of the variance in anxiety and correctly classified 81% of the cases. Female gender (*OR* 1.83; 95% CI 1.12–3.01), depression (*OR* 4.04; 95% CI 2.51–6.62), personal burnout (*OR* 1.69; 95% CI 1.04–2.76), TLRS (*OR* 1.74; 95% CI 1.11–2.73), and past history of mental illness (*OR* 2.57; 95% CI 1.09–6.48) were positively associated with anxiety. Whereas, being satisfied with academic performance (*OR* 0.60; 95% CI 0.38–0.93), getting adequate sleep (*OR* 0.52; 95% CI 0.31–0.88), being a second-year resident (*OR* 0.29; 95% CI 0.12–0.72), and being involved in extracurricular activities less frequently were negatively correlated with anxiety. This was more clearly seen by releveling the variable ‘Extracurricular involvement’ so that the reference class for dummy coding was ‘never’. In doing so, anxiety was positively predicted in students who were ‘always’ involving in extracurricular activities (β = 0.27, *OR* 1.31; 95% CI 0.21–6.61). But it was non-significant (*P* = 0.76).

**Table 7:** Final model of a multivariable logistic regression analysis predicting anxiety among medical students and residents

Predictor	β	***SE***	***P***	***OR*** (95% CI)
Intercept	1.051	0.534	0.049	2.860 (1.019–8.309)
Gender	–	–	–	–
Male ^a^	–	–	–	–
Female	0.604	0.251	0.016	1.830 (1.122–3.010)
Year of study	–	–	–	–
Med1 ^a^	–	–	–	–
Med2	−0.605	0.458	0.186	0.546 (0.220–1.331)
Med3	−0.075	0.411	0.855	0.928 (0.413–2.077)
Med4	−0.901	0.486	0.064	0.406 (0.154–1.043)
Med5	−0.628	0.463	0.175	0.534 (0.214–1.319)
Med6	−0.810	0.533	0.129	0.445 (0.153–1.248)
PGY1	−0.873	0.452	0.053	0.418 (0.170–1.005)
PGY2	−1.229	0.462	0.008	0.293 (0.116–0.716)
PGY3	−0.787	0.451	0.081	0.455 (0.186–1.096)
Depression	–	–	–	–
Absent ^a^	–	–	–	–
Present	1.396	0.247	< 0.001	4.039 (2.509–6.620)
Personal burnout	–	–	–	–
Absent ^a^	–	–	–	–
Present	0.526	0.247	0.033	1.693 (1.043–2.755)
Patient-related burnout	–	–	–	–
Absent ^a^	–	–	–	–
Present	0.594	0.312	0.056	1.810 (0.990–3.370)
Satisfied with academic performance	–	–	–	–
Dissatisfied ^a^	–	–	–	–
Satisfied	−0.516	0.225	0.022	0.597 (0.383–0.928)
TLRS	–	–	–	–
Absent ^a^	–	–	–	–
Present	0.555	0.229	0.015	1.743 (1.114–2.733)
Past history of mental illness	–	–	–	–
Absent ^a^	–	–	–	–
Present	0.943	0.450	0.036	2.568 (1.092–6.480)
Extracurricular involvement	–	–	–	–
Always ^a^	–	–	–	–
Often	−1.003	0.401	0.012	0.367 (0.165–0.799)
Sometimes	−0.761	0.340	0.051	0.467 (0.215–0.995)
Rarely	−1.317	0.446	0.003	0.268 (0.110–0.634)
Never	−0.268	0.862	0.756	0.765 (0.151–4.745)
Sleep adequacy	–	–	–	–
Inadequate sleep ^a^	–	–	–	–
Adequate sleep	−0.650	0.269	0.016	0.522 (0.306–0.882)
Test	χ^2^	*df*	*P*	
Hosmer and Lemeshow test	8.49	8	0.387	
Likelihood ratio test	128.22	20	*<* 0.001	

*Note.*^a^referent for dummy coding. *TLRS*, Teaching and Learning Related Stressor. Null deviance: 663.55 on 479 *df*, Residual deviance: 535.33 on 459 *df*, AIC: 577.33, AUC: 0.81

### Independent correlates of burnout-related symptoms

Many variables, that were associated with burnout symptoms, were shown by the univariate analysis (Table 5). Logistic regression was used to assess the impact of available variables on the likelihood that the study participant would suffer from burnout or not. The final model (Table 8) was statistically significant (*P* < 0.001), indicating that the model was able to distinguish whether the study participant was suffering from burnout or not. The final model explained between 26.2% (Cox and Snell R squared) and 35% (Nagelkerke R squared) of the variance in burnout and correctly classified 67% of the cases. Depression (*OR* 2.03; 95% CI 1.26–3.29), anxiety (*OR* 2.37; 95% CI 1.51–3.75), being a first-year resident (*OR* 2.38; 95% CI 1.02–5.69), DRS (*OR* 2.06; 95% CI 1.30–3.28), and a rare/never involvement in extracurricular activities were risk-enhancing correlates. Substance use was nearly the significant predictor of burnout (*P* = 0.06, *OR* 1.57; 95% CI 0.99–2.50).

**Table 8:** Final model of a multivariable logistic regression analysis predicting burnout among medical students and residents

Predictor	β	***SE***	***P***	***OR*** (95% CI)
Intercept	−2.595	0.684	< 0.001	0.075 (0.019–0.277)
Year of study	–	–	–	–
Med1 ^a^	–	–	–	–
Med2	−0.605	0.473	0.201	0.546 (0.211–1.362)
Med3	−0.370	0.402	0.357	0.691 (0.312–1.515)
Med4	−0.626	0.482	0.194	0.535 (0.204–1.359)
Med5	0.138	0.450	0.758	1.149 (0.475–2.782)
Med6	0.173	0.523	0.741	1.189 (0.423–3.312)
PGY1	0.868	0.437	0.047	2.382 (1.021–5.690)
PGY2	0.587	0.455	0.197	1.799 (0.741–4.438)
PGY3	−0.027	0.439	0.952	0.974 (0.411–2.309)
Depression	–	–	–	–
Absent ^a^	–	–	–	–
Present	0.709	0.243	0.004	2.031 (1.263–3.285)
Anxiety	–	–	–	–
Absent ^a^	–	–	–	–
Present	0.864	0.232	< 0.001	2.374 (1.510–3.753)
Satisfied with career choice	–	–	–	–
Dissatisfied ^a^	–	–	–	–
Satisfied	−0.499	0.262	0.057	0.607 (0.362–1.014)
Extracurricular involvement	–	–	–	–
Always ^a^	–	–	–	–
Often	0.591	0.413	0.152	1.806 (0.817–4.145)
Sometimes	0.449	0.401	0.263	1.567 (0.724–3.519)
Rarely	0.996	0.446	0.025	2.709 (1.145–6.618)
Never	2.324	1.165	0.046	10.215 (1.446–6.618)
ARS	–	–	–	–
Absent ^a^	–	–	–	–
Present	0.760	0.394	0.054	2.137 (1.009–4.768)
IRS	–	–	–	–
Absent ^a^	–	–	–	–
Present	0.412	0.245	0.092	1.511 (0.936–2.446)
DRS	–	–	–	–
Absent ^a^	–	–	–	–
Present	0.723	0.236	0.002	2.061 (1.299–3.282)
Substance use	–	–	–	–
Not using ^a^	–	–	–	–
Using	0.450	0.236	0.057	1.568 (0.989–2.502)
Test	χ^2^	*df*	*P*	
Hosmer and Lemeshow test	12.7	8	0.12	
Likelihood ratio test	145.9	19	< 0.001	

*Note.*^a^Referent for dummy coding. *ARS*, Academic Related Stressor; *IRS*, Interpersonal and intrapersonal Related stressor; *DRS*, Drive and Desire Related Stressor. Null deviance: 664.41 on 479 *df*, Residual deviance: 518.51 on 460 *df*, AIC: 558.51, AUC: 0.84

## Discussions

In the present study, the prevalence of depression, anxiety, and burnout among medical students and residents, and their possible association with predictor variables were assessed. Burnout was more prevalent than anxiety and depression in the study population: 48.8, 45.31, and 31.03% respectively. Residents suffered more burnout and depression (64.5 and 33.7%) than medical students (37.6 and 29.1%). However, medical students reported more anxiety symptoms compared to residents, 46.3% vs 44%. These data closely resembled with findings from other studies. For example, the prevalence of depression in medical students as seen in our study was similar to the global prevalence of depression among medical students (28%) as reported in a meta-analysis [36]. Similarly, a number of Nepalese studies [18–20] also reported similar prevalence of depression (29–30%) among medical students. The prevalence of depressed residents seen in our study was closer to the pooled prevalence of 28.8% reported by Mata et al. [69] in 2015. As only the medical students were included in the previous studies, we currently have no available data concerning resident physicians for comparison. The proportion of burnout seen among medical students in the study was slightly lower than the estimated prevalence of 44.2% burnout in a recently published meta-analysis [70]. The prevalence of burnout among residents in our study was higher than the aggregate prevalence (51%) reported by Low et al. [32] in their meta-analysis of the prevalence of burnout in medical and surgical residents. The proportion of anxious medicals students was slightly higher than what was reported in a previous Nepalese study (41.1%), which included medical students from two medical colleges [19]. However, anxiety seen among medical students in our study was much greater than what was reported in an Ethiopian study (30.1%) [71]. Consistent with previous studies [72, 73], academic-related stressors were causing high stress to the participants.

### Predictors of depression

The logistic regression model showed that depression, anxiety, and burnout were independently and strongly correlated with each other. Presence of one significantly predicted the occurrence of the other two. Studies done in different settings have found a significant association of burnout with depression [38, 46] and anxiety [46], consistent with our finding. In the same study [46], anxiety was identified as a significant predictor of depression.

Those who were satisfied with their academic performance were less likely to be depressed and anxious. This association between academic performace and depression has been reported in a similar Indian study in which 237 medical students were surveyed [74]. Knowing this, one might be curious about the effects of academic related matters on students’ mental health. We found that academic-related stressors (ARS) caused high-grade stress among study participants. ARS refers to educational or student events like examination systems, assessment methods, grading methods, acadmic schedule, getting poor marks in examination, high self expectation to do well in studies, large amount of content to be studied, having difficulty to understand content, lack of time to do revision, and having difficulty answering questions given by teachers [75]. In a 2018 study in western Nepal, more than 50% of second-year medical students were found to have suffered from academic stress [17]. Students who were dissatisfied with their academia would be less likely content with themselves, thus contributing to their stress, anxiety, and depression.

### Predictors of anxiety

Being a female was highly predictive of anxiety symptoms. Similar results were presented by Kebede et al. [71] in their study among medical students of Ethiopia. It might be because, males are less likely to disclose psychological suffering and seek help, compared to females [76]. However, gender was not a significant correlate for depression and burnout in our logistic model. The finding was in agreement with the finding of a meta-analysis of burnout among medical students [70]. In contrast, there were debating issues considering the effect of gender on depression among medical students. Many studies have found a significantly higher prevalence of depression in females [77–79], while a study in 1988 argued that female medical students were not more susceptible to depressive symptomatology than males [80].

Students who felt stressed with TLRS were more likely to feel depressed and anxious, which was in line with a previous review in which stress led to depression and anxiety [81]. TLRS relates to the teachers’ competency to teach and supervise students, the clarity of learning objectives delivered to students, and appropriateness of tasks given to students [75]. This might be conveying a message that the present way of teaching and learning activity is not being as friendly to students as it should be. A thorough change in the present curriculum might be necessary. Exceptional results have been seen by making changes in the results of examinations to pass/fail grading system [82].

Sleep duration is one of the important factors which determines sleep quality [83]. In our study, anxiety was unlikely in those getting adequate sleep. This matches well with several studies on sleep quality and psychological morbidity among university students. For instance, Lemma et al. [84] found a strong correlation of the symptoms of depression and anxiety with sleep quality. Poor sleep quality was also found to be associated with poor academic performance among medical students [85].

Second-year residents were less likely to suffer from anxiety symptoms than first-year medical students. The difficulty accommodating to the new learning environment might have contributed to anxiety in first-year medical students. Being more mature, residents, on the other hand, are expected to be less apprehensive than the younger medical students. Probably, second-year residents might be feeling relieved from their hectic first-year schedule. Many of the second-year resident physicians share their first year experience in which they frequently mention about their feeling of being overwhelmed by the tasks which were normally expected of them. During the second year, they might have an additional opportunity to delegate some of their tasks to the junior residents, probably reducing their perceived stress.

Someone who was frequently involving in extracurricular activities was more likely to develop anxiety. However, other studies are in disagreement with our results. Moor et al. [86] have found that exercise was associated with lower levels of anxiety, depression, and neurosis. Similarly, a reduction in anxiety sensitivity by both high-and low-intensity exercises was noticed by Broman-Fulks et al. [87]. However, we have not specified the type of extracurricular activities. Similarly, we have not sought out the motivation behind participation in extracurricular activities as they have different effects in the experience of burnout [88, 89].

### Predictors of burnout

Those who were less frequently involved in extracurricular activities were more likely to feel burnt out than others, though the association was not fairly significant. We feel the necessity to summarize some findings from other studies in this regard. Youssef [21] found that students who exercised regularly and had time to relax had lower rates of burnout. In contrast, a survey conducted among medical students in Saudi Arabia [90] found no significant association between burnout levels and the frequency of involvement in extracurricular activities. Similarly, to disagreement of our finding, Muzafar et al. [91] in their cross-sectional study of burnout among Pakistani medical students, have found that students who were less frequently involved in extracurricular activities had lower levels of burnout. It is possible that those students who were experiencing burnout might have used extracurricular involvement as a coping mechanism to lighten their feeling of burnout.

The first-year residents had significantly higher odds of being burnt out than others. The finding is consistent with a study in which a higher proportion of burnout was seen in first [38, 92] and second-year residents [92]. Another study reported that the second-year residents were less emotionally exhausted and had less psychosomatic symptoms compared to the time when they were in their first year [93]. The higher average age of enrollment of residents exposes them to different contextual and social factors, and pressures, which may influence their experience of burnout [94]. Besides, first year residents face higher work load in a new environment compared to their seniors. However, contrary to these findings, Michel et al. [95] have found a higher level of depersonalization among residents in their second and third years than they experienced in their first. What we feel is more important in our study is that residents were found more burnt out than medical students. Siu et al. [96] in their study among public doctors reached a similar conclusion. They have concluded that young and moderately experienced doctors who needed to work in shifts were more vulnerable to burnout. The median years of practice of doctors experiencing high-burnout were 8.5 (range 5–15) years. In our context, the residents tend to belong to this young and moderately experienced group of doctors. Other factors like increased paperwork, clinical audits, lack of autonomy, and overwhelming duty hours might have contributed to residents’ burnout in our setting. Besides, there is no advocacy to safeguard working conditions in our setting as in Europe [97] and the United States [98]. However, some studies contradicted our belief regarding duty hours in which no significant association was seen between the residents’ burnout with duty hours [99, 100]. Interestingly, in the same study [100], burnout was found to be strongly correlated with quantitative work overload. In a recent study in Japanese residents, long work hours were significantly associated with the development of depressive symptoms [101]. Nonetheless, a meta-analysis has proposed reduction in duty-hours as a measure to decrease burnout in residents [102].

The presence of a higher degree of DRS significantly predicted burnout. DRS arises from an unwillingness to study medicine due to various reasons such as the field of medicine not being one’s first priority, following friends to study medicine, parental wish to study medicine, wrongly choosing the specialty, and being demotivated after learning the reality of medicine [75]. The authors, unfortunately, came across a few circumstances in which some medical students became demotivated after living the reality of medicine. Most of them followed their parents’ wish or dreamt of their higher social status before enrolling into the medical school. Later, they got dissatisfied with their chosen career. In this study, we have also found that those who were satisfied with their career choice were less likely to develop burnout (*P* = 0.057).

In a study conducted in the Netherlands among preclinical medical students, less than six hours of sleep per night was well correlated with burnout [103]. Though sleep adequacy was significantly associated with the burnout symptoms in univariate analysis, it failed to contribute to the best-fitted model. Similarly, married students felt significantly higher burnout than others during univariate analysis but failed to show its significance in the overall model. Though some studies reported significantly higher burnout among unmarried residents [92], we think the situation in our setting is different. Looking at our context, the families of married participants are expecting financial support from them. They may find it difficult to fulfill the emotional demands of their families in addition to their academic obligation causative to their experience of burnout.

Some limitations of the study are acknowledged. Being a cross-sectional study, causal relationships between the associations could not be established. So, multicenter studies with longitudinal follow-up design are still required to elucidate the course of depression, anxiety, and burnout. The levels of the outcome variables might be high due to ongoing exams of medical students. The statistical power may have been reduced as some variables were dichotomized. The variables that might be related to depression such as personality characteristics [104], social support systems, and the ongoing conflict between career life and personal life were not analyzed. We have not sought the frequency of consumption of abusive substances. The choice of response and the degree of honesty in disclosing one’s problems might have been affected by the level of stigma in our setting. Similarly, we have not included information about specialty among residents. As the study was conducted in a single medical school, this may not be representative of the national outcomes of depression, anxiety, and burnout in medical students and residents. Since we have used screening tools in this study, confirmation of the findings of this study requires further clinical psychiatric diagnostic interview through the use of a structured psychiatric diagnostic instrument.

## Conclusions

High prevalence of depression, anxiety, and burnout were seen among medical students and residents, with the predominant effect of high-grade academic-related stressors. The study will provide baseline data for future multicenter prospective studies. The researcher hopes that this will help the relevant authorities to effectively design strategies for mental well-being of future physicians. The strong correlation of TLRS with anxiety may be calling for more student-friendly curriculum. This can be achieved after a significant change in the present curriculum without undermining the objectives of medical learning.

## Supplementary information

**Additional file 1.**

## Data Availability

The datasets used and analyzed during the current study, and the scripts used during multivariable logistic regression analysis in R-software environment are available from the corresponding author upon reasonable request.

## Abbreviations

ARS: Academic Related Stressor
AIC: Akaike Information Criterion
AUC: Area Under Receiver Operating Characteristic Curve
CBI: Copenhagen Burnout Inventory
DRS: Drive and Desire Related Stressor
GARS: Group Activities Related Stressor
HADS-A: Hospital Anxiety and Depression Scale-Anxiety
HADS-D: Hospital Anxiety and Depression Scale-Depression
IRS: Interpersonal and Intrapersonal Related Stressor
MSSQ: Medical Students’ Stressor Questionnaire
SRS: Social Related Stressor
TLRS: Teaching and Learning Related Stressor
VIF: Variance Inflation Factor
