# Supplementary material for: Depression, anxiety, and burnout among medical students and residents of a medical school in Nepal: a cross-sectional study
Source: BMC Psychiatry. 2020 Jun 15;20:298. doi: 10.1186/s12888-020-02645-6 (PMC7294639; doi:10.1186/s12888-020-02645-6)
Supplement: Supplementary file 1 — Additional file 1. [file 12888_2020_2645_MOESM1_ESM.pdf]

Depression, anxiety and burnout among medical students and residents of a medical school in Nepal

Consent form

Dear participant, we invite you to participate in a research study entitled "**Depression, anxiety and burnout among medical students and residents of a Medical** School in **Nepal."** The purpose of this research is to determine the prevalence of above-mentioned mental health problems among medical students and residents. The enclosed questionnaire has been designed to collect symptoms of depression, anxiety and burnout including information on your sociodemographic profile.

Your participation in this research is completely voluntary. You may decline altogether, or leave blank questions you do not wish to answer. There are no known risks of discomforts associated with this survey. Your responses will be kept strictly confidential, and digital data will be stored in secure computer files after it is entered. Data from this research will be reported only as a collective combine total. Any report of this research that is made available to the public will not include your name or any other individual information by which you could be identified.

If you agree to participate in this study, please answer the questions on the questionnaire as best as you can. It should take approximately 15 minutes to complete. Please return the questionnaire as soon as possible. If you have any questions about this project, feel free to contact Nishan Babu Pokhrel at nishanpokhrel1@iom.edu.np. Information on the rights of human subjects in research is available through the Institutional Review Board of Institute of Medicine. Email: irb@iom.edu.np.

Completing this survey indicates that you are 18 years of age or older and that you have provided your consent to participate in this research.

Thank you for your assistance in this important endeavor.

Yours.

Nishan Babu Pokhrel, MBBS (Principal Investigator)
Ramesh Khadayat, MBBS (Co-principal investigator)

Please take a few minutes to fill out this survey. Your answers will be kept confidential. Thank you for the participation.

	**SECTION I**		
	**Socio-demographic information**		
1.<br>2.<br>3.	Age:<br>Gender: ☐ Male ☐ Female<br>Year of medical school: ☐ First ☐ second ☐ Third ☐ Fourth year ☐ Final year ☐ Intern ☐ Resident (_____ year)<br>If you are resident, please mention whether you are: ☐ Clinical ☐ Non-clinical/ Basic science		
4.<br>5.	Were you enrolled fresh in this medical school?<br>☐ yes ☐ No<br>**If not,** how many years have been lost after 10+2/ISc before you were enrolled in this medical school? ____________years.		
6.	Religion: ☐ Hindu ☐ Buddhism ☐ Muslim ☐ Christian ☐ Others (please specify) ____________		
7.	Nationality: ☐ Nepali ☐ Indian ☐ Others (Please specify) ______________		
8.	Where do you stay? ☐ At home ☐ Rented room ☐ Hostel ☐Others (specify) ____________		
9.	Relationship status: ☐ Married ☐ Non-married partner ☐ Single ☐ Divorced		
10.	Children: ☐ Yes (no of children)______☐ None		
11.	What is the average monthly income of your **FAMILY**?	☐ ≥ 46,000 (NRs)<br>☐ 23, 000- 46, 000 (NRs)<br>☐ 17, 000- 23, 000 (NRs)<br>☐ 11, 000- 17, 000 (NRs)<br>☐ 7, 000- 11, 000 (NRs)<br>☐ 2,000- 7, 000 (NRs)<br>☐ ≤ 2, 000 (NRs)	
	**SECTION II**		
12.	Do you smoke *cigarettes?*	☐ YES	☐ NO
	If you check "**NO**" then continue to question 25.		
13.	When did you start *smoking?*	☐ Before joining medical school<br>☐ After joining medical school	
14.	If you started smoking before joining medical school, did the frequency increase or decrease after joining medical school?	☐ Increase	☐ Decrease
15.	Do you smoke *marijuana?*	☐ YES	☐ NO
	If you check "**NO**" then continue to question 28.		
16.	When did you start using *marijuana?*	☐ Before joining medical school<br>☐ After joining medical school	

17.	If you started using marijuana before joining medical school, did the frequency increase or decrease after joining medical school?	☐ Increase	☐ Decrease
18.	Do you drink *alcohol?*	☐ YES	☐ NO
	If you check “**NO”** then continue to question 31.		
19.	When did you start drinking *alcohol?*	☐ Before joining medical school<br>☐ After joining medical school	
20.	If you started using alcohol before joining medical school, did the frequency increase or decrease after joining medical school?	☐ Increase	☐ Decrease
21.	Have the thoughts of *committing suicide* came in your mind while in medical school?	☐ YES	☐ NO
22.	Have you considered *dropping out* of medical school in past month?	☐ YES	☐ NO

23. SECTION III: Please tick (✓) in the box below, for which you feel is the most appropriate answer.

**** If you are not in clinical year yet, please answer based on what do you feel if you face this situation***

**Questions**		**Answers**				
		Always	Often	Sometimes	Seldom /Rarely	Never/Almost never
1	How often do you feel tired?					
2	How often are you physically exhausted?					
3.	How often are you emotionally exhausted?					
4.	How often do you think: “I can’t take it					
5.	How often do you feel worn out?					
6.	How often do you feel weak and susceptible to					
7.	Do you feel worn out at the end of the					
8.	Are you exhausted in the morning at the thought of another day at work/study?					
9.	Do you feel that every working/studying hour is					
**10.**	**Do you have enough energy for family and friends during leisure time?**					
18.	Are you tired of working with patients?					
19.	Do you sometimes wonder how long you will be					
		To a *very* high degree	To a high degree	Somewhat	To a *low* degree	To a ***very*** *low* degree
11.	Is your work/study emotionally exhausting?					
12.	Does your work/study frustrate you?					
13.	Do you feel burnt out because of your					
14.	Do you find hard to work with patients?					
15.	Does it drain your energy to work with patients?					
16.	Do you find it frustrating to work with patients?					
17.	Do you feel that you give more than you get back when you work with patients?					

24. SECTION IV

From the listed items below, please rate them according to the following:
0 = causing no stress at all, **1** = causing mild stress, **2** = causing moderate stress, **3** = causing high stress, **4** = causing severe stress

If you are not in clinical year yet, please answer based on what do you feel if you face this situation.

	**Items**	**How do you rate it?**				
		**0**	**1**	**2**	**3**	**4**
1	Tests/examinations					
2	Falling behind in reading schedule					
3	Large amount of content to be learnt					
4	Lack of time to review what have been learnt					
5	Heavy workload					
6	Participation in class presentation					
7	Need to do well (imposed by others)					
8	Feeling of incompetence					
9	Unable to answer questions from patients*					
10	Talking to patients about personal problems*					
11	Facing illness or death of the patients*					
12	Verbal or physical abuse by other student(s)					
13	Verbal or physical abuse by teacher(s)					
14	Verbal or physical abuse by personnel(s)					
15	Conflict with teacher(s)					
16	Unwillingness to study medicine					
17	Parental wish for you to study medicine					
18	Not enough feedback from teacher (s)					
19	Uncertainty of what is expected of me					
20	Lack of recognition for work done					

Verbal Abuse is defined as to speak insultingly, harshly and unjustly about a person.

Physical Abuse is defined as to treat in harmful, injurious or offensive way to a person.

25. Section V: Please tick (✓) the most appropriate option.

Tick the box beside the reply that is closest to how you have been feeling in the past week.
Don't take too long over you replies: ***your immediate is best.***

A_1- म आफूलाई तनावमा पाउँछु:<br>३. प्राय:जसो<br>२. धेरै पटक<br>१. समय समयमा<br>०.कहिल्यै पनि हुन्नँ	D_4- म शिथिल भएको महसुस गर्दछु:<br>३. प्राय: सँधैजसो समय<br>२. बारम्बार<br>१. कहिलेकाहीं<br>०. कहिल्यै पनि हुन्नँ
D_1- म अझै पनि आफूलाई मन पर्ने चीजमा खुशी पाउँछु:<br>०. पक्कै पनि धेरैपटक<br>१. त्यत्ति धैरे होइन<br>२. अलि अलि<br>३. कहिल्यै पनि हुन्नँ/बिरलै	A_5- म डर लागेजस्तो भएर मन सिरिङ्ग गरिरहेको अनुभव गर्दछु:<br>०. कहिल्यै पनि होइन<br>१. कहिलेकाहीं<br>२. अत्यधिक मात्रामा<br>३. सँधैंजसो
A_2- म केही डरलाग्दो घटना घट्ला कि भनेर डराउँछु:<br>३. एकदमै नराम्रो सँग<br>२. हो, तर त्यति नराम्रोसँग होइन<br>१. अलि अलि तर त्यत्ति चिन्तित गराउँदैन<br>०. पटक्कै लाग्दैन	D_5- मेरो आफ्नो रंगरुपमा रुचि घटिरहेको छ:<br>३. अवश्य नै<br>२. जति लिनुपर्ने हो त्यति लिन्नँ<br>१. त्यत्ति रुचि नदिन सक्छु<br>०. पहिले जस्तै चासो राख्छु
D_2- म कुनै पनि कुराको रमाइलो पक्षलाई देखेर हाँस्न सक्छु:<br>०. पहिले जत्ति नै<br>१. अहिले त्यत्ति धेरै होइन<br>२. पक्कै तर पहिले जस्तो होइन<br>३. हुँदै हुन्नँ	A_6- म सधैं छटपटी/अशान्त जस्तो अनुभव गर्दछु:<br>३. वास्तवमा धेरै नै<br>२. अलि बढी<br>१. त्यत्ति बढी होइन<br>०. हुँदै होइन
A_3- चिन्ताजनक विचारहरु मेरो दिमागमा आउँछन्:<br>३. अत्यधिक मात्रामा<br>२. धेरैजसो<br>१. कहिलेकाहीं तर त्यति धेरै आउँदैन<br>०. बिरलै	D_6- म जहिले पनि रमाइलो गर्न मनोरञ्जनको प्रतीक्षामा हुन्छु:<br>०. पहिले जस्तो नै<br>१. पहिले भन्दा अलि कम<br>२. पक्कै पनि पहिले भन्दा थोरै<br>३. कहिल्यै पनि होइन
D_3- म आफूलाई सन्तुष्ट अनुभव गरिरहेको छु:<br>३. कहिल्यै पनि हुन्नँ<br>२. प्राय: हुन्नँ<br>१. कहिलेकाहीं<br>०. धेरैजसो समय	A_7- म कहिलेकाहीं एकदमै अत्तालिन्छु:<br>३. वास्तवमा धेरै नै<br>२. प्राय: जसो<br>१. त्यति धेरै होइन<br>०. हुँदै होइन
A_4- म ढुक्क एवं निश्चिन्त बस्न सक्छु:<br>०. पक्कै पनि/अवश्य<br>१. प्राय:जसो<br>२. कहिलेकाहीं<br>३. हुँदै हुन्नँ	D_7- म गफगाफ, रेडियो, किताब, टि.भी, भिडियोजस्ता कार्यक्रममा रमाउँछु:<br>०. प्राय:<br>१. कहिलेकाहीं<br>२. त्यत्ति धेरै होइन<br>३. बिरलै

Please check you have answered all the questions.

SECTION VI

	**Personal information**		
26.	Relationship problem with family/friends	☐ YES	☐ NO
27.	Did you suffer from mental disorders in the past?	☐ YES	☐ NO
28.	Have you consulted the specialist regarding this?	☐ YES	☐ NO
29.	Would you like to consult specialist regarding your problem?	☐ YES	☐ NO
30.	Are you currently receiving treatment for the mental health issue?	☐ YES	☐ NO
31.	History of mental disorders in family:	☐ YES	☐ NO
32.	Are you satisfied with your academic performance?	☐ YES	☐ NO
33.	Are you satisfied with your career choice?	☐ YES	☐ NO
34.	How frequently do you involve in extracurricular activities?	☐ Always<br>☐ Often<br>☐ Sometimes<br>☐ Rarely<br>☐ Never	
35.	How many hours do you sleep in an average day?	_____________hours	
36.	What is the educational level of the head of your family?	☐ Professional or honors<br>☐ Graduate or post graduate<br>☐ Intermediate<br>☐ High school certificate<br>☐ Middle school certificate<br>☐ primary school certificate	
37.	What is the occupation of the head of your family?	☐ Profession<br>☐ Semi profession<br>☐ clerical, shop owner or farmer<br>☐ skilled owner<br>☐ semi-skilled worker<br>☐ Unskilled worker<br>☐ Unemployed	
